# The Role of Primary School Composition in the Trajectories of Internalising and Externalising Problems across Childhood and Adolescence

**DOI:** 10.1007/s10802-019-00584-9

**Published:** 2019-09-21

**Authors:** Efstathios Papachristou, Eirini Flouri, Emily Midouhas, Glyn Lewis, Heather Joshi

**Affiliations:** 1 Department of Psychology and Human Development, UCL Institute of Education, University College London, 25 Woburn Square, WC1H 0AA, London, UK; 2 Division of Psychiatry, University College London, London, UK

**Keywords:** England, Externalising problems, Internalising problems, Millennium cohort study, School composition, School effects

## Abstract

There is little research on the role of school and its composition in explaining individual children’s psychological outcomes. This study examined for the first time the role of several primary-school compositional characteristics, and their interactions with individual level characteristics, in the development of two such outcomes, internalising and externalising problems, at ages 7, 11 and 14 years in 4794 children in England participating in the Millennium Cohort Study. Using hierarchical (multilevel) linear models, we found that, even after adjusting for individual and family characteristics, children in schools with higher proportions of pupils eligible for free school meals had more externalising problems. In general, children with special educational needs, lower academic performance, more distressed mothers, and those in non-intact families had more internalising and externalising problems. Our results underline the importance of targeting schools with less affluent overall intakes, but also highlight the key role of individual and family characteristics in the development of their pupils’ psychological functioning.

## Introduction

School is considered the most important extra-familial context for children. They spend great amounts of time in schools, where they share teachers and social norms, and interact with peers. There is some evidence for the positive role of certain characteristics of this context in children’s health outcomes. Such characteristics include a higher socio-economic status (SES) intake, small classes, good teachers and effective classroom and school management which allow for better and more individualised instruction (Bonell et al. 2013; Milkie and Warner 2011; Richmond and Subramanian 2008; Sellström and Bremberg 2006; West et al. 2004). There is also evidence that these school characteristics can affect indirectly children’s and adolescents’ psychological outcomes. For example, they can impact on individual children’s academic performance (Konstantopoulos and Borman 2011; Nye et al. 2004; Rumberger and Thomas 2000), which is, in turn, associated with self-esteem and academic self-concept (Cvencek et al. 2018) as well as low levels of internalising and externalising problems (Van der Ende et al. 2016). However, there is relatively little research on the direct role of schools and their characteristics in explaining differences in individual pupils’ mental health. In this study we sought to fill this gap by examining the role of primary school composition in the levels of internalising and externalising problems from mid-childhood to mid-adolescence (ages 7 to 14 years). Building on evidence suggesting that the developmental trajectories of these problems can vary substantially across childhood and adolescence (Flouri et al. 2018), we also aimed to explore the role of primary school composition in dampening, or conversely, exacerbating the influence of individual characteristics on these trajectories.

To date, the main school compositional characteristics that have been associated with key outcomes in youth are ethnic density and ethnic diversity (both related to ethnic composition), academic performance and SES. School-level ethnic diversity (heterogeneity) is typically quantified as the probability that two randomly selected pupils from the same school are of different ethnic origin (Benner and Yan 2015). Benner and Crosnoe (2011), who examined the roles of within-school ethnic diversity and ethnic density [also known as ethnic congruence; the proportion of co-ethnics in a school (Benner and Graham 2007; Fleischmann et al. 2012)] in children’s outcomes at school entry, showed cognitive benefits for ethnic diversity and emotional benefits for ethnic density (Benner and Crosnoe 2011). They, and others (Gurin et al. 2004), argue that in many ways diversity provides opportunities for valuable cognitive exercise, and should therefore promote academic outcomes; cognitive growth is fostered when individuals encounter experiences and demands that they cannot completely understand or easily meet, such as those brought about by interactions with peers from other ethnicities (Pickett and Wilkinson 2008). Ethnic density, on the other hand, is generally associated with higher levels of social support and a reduction of exposure to racism by dispelling prejudices. For example, reviewing research examining the role of community (neighbourhood) ethnic composition, Shaw et al. (2012) report that members of most ethnic minority groups have better mental health when they live in neighbourhoods with higher proportions of people of the same ethnicity, a phenomenon termed the ‘ethnic density effect’ and seen in educational contexts too (Gieling et al. 2010).

With respect to school-level academic performance, it seems that children attending lower-performing schools have higher levels of behavioural problems, such as delinquent behaviour (Dudovitz et al. 2018; Wong et al. 2014) and emotional symptoms (E. Goodman et al. 2003), for two main reasons. First, schools promoting academic achievement foster behaviours and habits that may lead to an improved future outlook and less risk taking (Kelly et al. 2005). Second, school-level performance is usually a good proxy for school culture and school connectedness (or school belonging) linked, respectively, to lower levels of externalising and internalising problems. A supportive school culture can isolate children from deviant peers in other schools while also altering opportunities and motivations to engage in risky behaviours by facilitating and enforcing positive peer interactions (Dudovitz et al. 2018). School connectedness, on the other hand, can protect from the effect of risk factors for depression, such as poor family relationships and negative life events (Millings et al. 2012), although with some caveats. Anderman (2002), for example, showed that aggregate school belonging was related positively to individual pupils’ academic achievement, but also, counterintuitively, to self-reported social rejection and academic and social problems at school. He suggested (but did not test) that in schools in which many pupils feel that they do belong, those who do not belong may experience more social rejection and problems in school.

Finally, regarding SES, there is a well-documented association between various indicators of socioeconomic disparity at the individual level and child and adolescent mental health (Reiss 2013). However, despite some evidence for an association between school-level poverty and emotional and behavioural problems in childhood (Flouri and Midouhas 2016; Midouhas 2017), the relationship between school-level SES and child and adolescent mental health remains largely unclear. Studies drawing upon ecological theories to examine the importance of individual and school-level SES in school misbehaviour, crime and misconduct (Stewart 2003; Wilcox et al. 2006) identify different effects for individual and contextual SES. Stewart (2003), for example, found a significant effect for individual, but not school level, poverty on misbehaviour, but suggested that economic inequality within the school, rather than school-level SES, might be a better predictor of misbehaviour. Such a pattern of relationships would be in line with predictions from, and findings in line with, the theory of relative deprivation (Stouffer et al. 1949). According to this theory, being relatively deprived in comparison to a reference group causes stress (Winkleby et al. 2006), which can in turn affect health negatively (Yngwe et al. 2003). A 2012 meta-analysis of the impact of relative deprivation on a range of outcomes provided conclusive evidence that one’s perception of their relative injustice compared to a well-defined reference group –school, neighbourhood, or other- can impact significantly on mental health (Smith et al. 2012). In educational settings, the Big-Fish-in-Little-Pond effect (Marsh and Hau 2003) similarly predicts that in contexts where social comparisons result in negative self-evaluations, self-concept suffers (Dicke et al. 2018). However, there is also evidence for the reverse. For example, Martens et al. (2014) who examined the effect of similar cross-level interactions on health and education outcomes found that, as expected, poor children overall fared worse than their less poor counterparts. However, neighbourhood-level poverty had a moderating role in that relationship, such that poor children in wealthier neighbourhoods had better outcomes, at least in some of the domains examined, in adolescence. This pattern of relationships is in line with an alternate theoretical model, also motivating several studies on compositional effects, the collective resources model (Macleod and Davey Smith 2003; Pearce and Davey Smith 2003; Stafford and Marmot 2003). According to this, social inequalities in outcomes do not stem from one’s relative position in the social hierarchy, but rather from absolute material deprivation. This is turn suggests that poor individuals in poorer contexts do worse than their counterparts in less poor contexts because their lack of resources at the individual-level combines with deprived social resources and neglected infrastructure at the community-level. There are of course other, non-causal, explanations for the association between school-level SES and individual pupils’ mental health. For example, schools with higher proportions of disadvantaged pupils have higher rates of bullying and victimisation (Jansen et al. 2012), greater exposure to parental mental illness and less parental involvement, which are all, in turn, associated with mental ill-health (Arseneault et al. 2010; Flouri and Buchanan 2003; Wang and Sheikh-Khalil 2014).

Regardless, even when seen as non-causal, the relationship between school’s social context and individual pupils’ outcomes is usually interpreted through a sociological lens. However, another theoretical framework that can be used to understand this relationship is provided by Bronfenbrenner’s bioecological model and Sameroff’s transactional model (Bronfenbrenner 1979; Bronfenbrenner and Morris 1998; Sameroff 1975). Bronfenbrenner conceptualised ecological systems that describe different aspects of an environment, each nested within the others, which interact to influence a child’s growth and development. The main systems include a) the child herself and what she brings to the world with her, e.g. personality characteristics –termed micro system; b) her immediate settings, e.g. family –termed meso system; c) her more distal settings, e.g. the neighbourhood –termed exo system; and d) the general society in which she lives –termed macro system. Similarly, Sameroff (1975) posited that development is driven by the complex interplay between a child’s inherent characteristics, family characteristics and her economic, social and community resources.

The common underlying theme of both models is that they consider outcomes to be driven by neither the individual nor the context alone; instead, outcomes are seen as the product of the relative and interactive effects of both individual and contextual factors. In addition, both models distinguish between interactions of factors that are in a child’s immediate environment (termed proximal processes or influences) from those affecting the child less directly (distal processes or influences). While proximal processes are the primary mechanism for development, both models emphasise both the importance of interactions between the various systems and the impact of distal influences on proximal processes, which in turn shape development. Researchers studying pupil outcomes using either model as a conceptual tool should thus be expected to examine the role of interactions between systems but also, importantly, how distal (e.g., school) processes can drive more proximal processes, in turn determining outcomes. To an extent this has been done in studies that, motivated by such ecological models, examined the role of school-level characteristics in academic and social outcomes (Benner et al. 2008; Benner and Yan 2015). For example, Benner and Yan (2015) found that greater classroom ethnic diversity promotes parental involvement, which is in turn associated with children’s interpersonal skills and reading achievement. Earlier Benner et al. (2008) demonstrated that structural characteristics of families and schools, including living arrangements, school-level SES, school size, and others, influenced proximal processes within each of these settings, which in turn, influenced academic attainment.

An additional key system in Bronfenbrenner’s bioecological model which was considered more recently (Bronfenbrenner and Morris 2006) is the chrono system, which encompasses the dimension of time. Elements within this system can be either external, such as the timing of a parent’s death, or internal, such as the normative changes that occur with the ageing of a child. This element of the model is particularly relevant for the study of pupil mental health across childhood and adolescence because the transition from childhood to adolescence is a critical developmental period (Sawyer et al. 2018). Importantly, the transition to adolescence coincides with another important transition: the one to secondary school. However, to our knowledge, this dimension is yet to be incorporated fully in studies applying an ecological lens on the relationship between school characteristics - and their interactions with the microsystem - and pupil mental health. Considering this element explicitly was an additional contribution of this study.

## The Present Study

In light of this literature, we explored the role of primary school composition in children’s trajectories of internalising and externalising problems from mid-childhood to mid-adolescence in England. We also investigated its role in changing children’s likelihood of following the trajectories expected on the basis of their individual and family characteristics. The features of school composition we considered were ethnic diversity, school-average academic performance [measured with Key Stage 1 (KS1) scores], as well as the proportion of children with free school meal (FSM) eligibility (as a proxy of SES), English as an additional language, and special educational needs (SEN). Eligibility for FSM is much used in UK research as a proxy for SES, as it reflects family eligibility to income tested benefits (Ilie et al. 2017); pupils are entitled to FSM if their parents receive certain means-tested benefits or tax credits subject to a gross household income ceiling. We tested cross-level interactions between a) school-average KS1 performance b) proportion of pupils in the school eligible for FSM and c) proportion in the school with SEN, and the equivalent individual characteristics (i.e. child’s KS1 score, child’s FSM eligibility and child’s SEN status) on the children’s trajectories. We expected that less disadvantaged socio-economic intake and less ethnic diversity would be associated with lower levels of both internalising and externalising problems in childhood. Regarding cross-level interactions, we expected that low-SES children (those eligible for FSM) in high-SES schools (schools with low proportion of children with FSM eligibility) would have more internalising problems.

## Methods

### Sample

We used data from the Millennium Cohort Study (MCS), a population-based cohort of children born in the UK over 12 months from 1 September 2000 (Joshi and Fitzsimons 2016). The children in the MCS were around 9 months old at Sweep 1, and 3, 5, 7, 11 and 14 years old at Sweeps 2–6, respectively. At the six sweeps, the numbers of productive families were 18,522, 15,590, 15,246, 13,857, 13,287 and 11,714. Ethical approval was gained from NHS Multi-Centre Ethics Committees. Parents gave informed consent before interviews took place, and from age 11 children gave assent.

When the children were aged 7, information was also collected from the cohort children’s class teachers using a self-completion postal questionnaire. In total, 7235 teachers in 4969 schools were contacted to take part in the survey. Of those, 5364 teachers (74.1%) from 3981 schools (80.1%) completed and returned a questionnaire for 8876 children. Ethical approval for the teacher survey was given by the Northern and Yorkshire Multi-Centre Research Ethics Committee (MREC) of the NHS (Huang and Gatenby 2010). Further approvals were sought and given for carrying out the survey in schools in each UK country. For England, the survey was approved by the Star Chamber in the Department for Children, Schools and Families; for Wales, by the Schools Workforce Advisory Panel; for Scotland, by the Directors of Education in the Local Educational Authorities; and for Northern Ireland, no formal approval was needed.

**Fig. 1:**
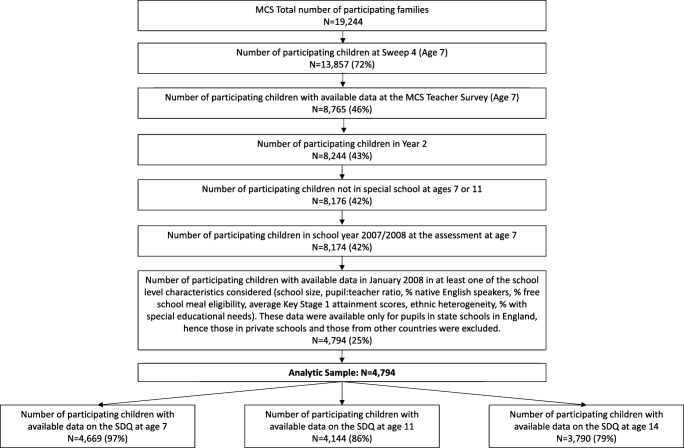
Flow chart of the study

We used data from sweeps 4 (age 7) to 6 (age 14). The flow chart illustrates the process followed to derive the analytic sample of this study (*n* = 4974; we count only one child per family, i.e., singletons and first-born twins or triplets) and associated attrition (Fig. 1). Our analytic sample included those with available information on at least one of the school compositional and structural characteristics considered at age 7, and all school information was obtained from the School Data Unit at the Department for Education. Since that information was about pupils in state schools in England, those from the other three UK countries as well as those in private schools had to be excluded. KS1 assessment data in England are collected at the end of Year 2 (normally the year in which pupils reach age 7) and therefore we also excluded two children who were not in Year 2 during that academic year. Finally, we excluded children attending special schools at ages 7 or 11.

## Measures

### Internalising and Externalising Problems

Internalising and externalising problems are the two main dimensions that have been widely used to characterise the structure of mental health in children and adolescents (Cicchetti and Toth 2014). Available evidence suggests that they are related to several common psychiatric disorders, including depression, anxiety disorders, substance use disorder, and antisocial personality disorder (Krueger et al. 2001). We assessed internalising and externalising problems in MCS using the parent-reported Strengths and Difficulties Questionnaire (SDQ) at ages 7, 11 and 14 years. The SDQ is a short, psychometrically-valid and widely-used behavioural screening tool (R. Goodman 1997). It comprises 25 items which form five scales: emotional symptoms, conduct problems, hyperactivity/inattention, peer problems and prosocial behaviour. In line with recommended practice for community samples (A. Goodman et al. 2010), the internalising problems scale comprises the 10 items from the emotional and peer problems subscales, and the externalising problems scale the 10 items from the hyperactivity and conduct problems subscales. Scores on the internalising and externalising problems scales range from 0 to 20 with higher scores indicating more serious problems or symptoms. The sum of the two scales yields a total difficulties score. In our analytic sample both internalising and externalising problems scales showed satisfactory internal consistency (Cronbach’s alpha values were 0.71 and 0.79, 0.77 and 0.80, and 0.78 and 0.81, at ages 7, 11 and 14, respectively).

### School Compositional Characteristics

School compositional characteristics obtained from the School Data Unit at the Department for Education applied to state-maintained schools during January 2008 (corresponding with MCS sweep 4, age 7) and were banded into deciles based on all primary schools in England. *School-average KS1 scores* were averaged for English, Maths and Science across pupils. School socio-economic composition was based on the percentage of pupils who were eligible for *FSM*. Additional school characteristics included the percentage of pupils whose *first language was known or believed to be English* and the percentage of children with *SEN* (without statements; a statement of SEN sets out the child’s needs and the help they should receive. It is reviewed every year to make sure that any extra support given meets the child’s needs). Finally, we created a census-based measure of school-level *ethnic heterogeneity* using Greenberg’s diversity index (Greenberg 1956), which utilises a formula that measures the probability that two randomly chosen individuals from a population in a given area will have the same ethnicity. All sensitive data were linked with MCS data in a secure environment using the unique anonymised reference number of each child’s school.

### Covariates

Information on individual *academic performance* (measured with the child’s KS1 average score) and *FSM eligibility* was collected during the January 2008 census and obtained from the National Pupil Database. We also adjusted for individual and family-level characteristics (at baseline, age 7 years) that are known to be associated with both selection into schools and psychological functioning in youth: *age* (in months), *gender, SEN status, ethnicity* (the standard eleven Census categories as listed in Table 3), *maternal education* (university degree or not), *family structure* (living with both biological parents or not) and *maternal psychological distress* [using the Kessler K6 (Kessler et al. 2002)] (Flouri et al. 2018). The K6 is a validated six-question instrument that was developed to estimate the prevalence of serious mental illness in general population samples (Kessler et al. 2010). In MCS, mothers were asked how often during the past 30 days they felt: ‘so depressed that nothing could cheer you up’, ‘hopeless’, ‘restless or fidgety’, ‘that everything was an effort’, ‘worthless’ and ‘nervous’. Each item is scored on a five-point Likert scale, from ‘None of the time’ (0) to ‘All of the time’ (4).We also considered two structural school characteristics, the headcount of pupils - as a measure of *school size* - and the *pupil:teacher* ratio which was computed by dividing the pupil headcount by the number of teachers.

### Statistical Analysis

First, we examined the distribution of internalising and externalising problems and the school, individual and family characteristics of the analytic sample at baseline (age 7). Next, we ran a sample bias analysis to examine differences in individual and family characteristics between children included in the analytic sample (*N* = 4794) and the non-analytic sample (*N* = 3380). We then ran a series of multilevel linear models (MLMs) to examine the associations of school characteristics at age 7 with trajectories of internalising and externalising problems at ages 7, 11 and 14 years. By running MLMs rather than simple fixed effects regression models we accounted for the hierarchical nature of the data with repeated measures (at ages 7, 11 and 14 years) of internalising and externalising problems for children, i.e. by having occasions (level 1) nested in children (level 2), thus obtaining more robust standard errors for the regression coefficients. We did not consider for this analysis (although we did perform a supplementary analysis, see under Sensitivity Analyses below) a third level (children nested within schools) in the MLMs because the degree of clustering of MCS children within school was not adequate. In the analytic sample, the majority of schools (66%) provided information for a single MCS child, 14% of schools for two MCS children and only 20% of schools for three or more, with the average MCS pupil count within a single school being 2.1.

In the first set of MLMs (Model A), we examined the crude associations between school compositional and structural characteristics with the trajectories of externalising and internalising problems. In the next set (Model B), we adjusted further for the individual and family covariates. We tested for multicollinearity between the covariates by using variance inflation factor (VIF) values [VIF values>4 indicate multicollinearity (Fox 2016)]. In the third set of analyses (Model C), we further examined the effect of cross-level interactions on the trajectories of externalising and internalising problems for those characteristics that were measured at both school and child levels; these included FSM eligibility, SEN status and KS1 score. We did not estimate cross-level interactions for English as an additional language on the trajectories of internalising and externalising problems because very few MCS children were reported to be non-English speakers at age 7 (all MCS children were born in the UK). Similarly, we did not estimate cross-level interactions between individual and school-level ethnicity because the variables used to capture school-level ethnic density had too many missing values to be reliably imputed (>50%). Although there were many cases where unmeasured school effects could not be disentangled from individual child characteristics due to the low degree of clustering of pupils within schools, we did attempt to estimate random effects for schools in a sensitivity analysis (reported below). For this analysis we tested the proportion of variance in the outcomes that is explained by commonalities in children attending the same school by introducing a 3rd level (children nested within schools) to the MLMs.

Missing data on outcome measures and covariates in the analytic sample were handled using multiple imputation by chained equations (Azur et al. 2011). We generated 20 imputed datasets using linear, logistic and ordered regressions depending on the scale of measurement of the variables being imputed, and performed complete-data analyses using Rubin’s combination rules to consolidate the obtained individual estimates into a single set of multiply imputed estimates. All MLMs took into account the stratified sample design of MCS by including the eight strata (England-disadvantaged, Wales-advantaged, Wales-disadvantaged, Scotland-advantaged, Scotland-disadvantaged, Northern Ireland-advantaged, and Northern Ireland-disadvantaged) as dummy variables (England-advantaged was the reference category and hence excluded) in the fixed part of the models [although the analytic sample were all observed in England at age 7, a small number of cases (*N* = 64) had moved into England from the other countries since the original sampling and hence were included in the fixed part of the MLMs with their varying initial sample probabilities]. Age was grand mean centred (at 127.79 months, i.e. 10.6 years) in all MLMs. Where appropriate, i.e., for those models in which significant associations between school compositional characteristics and the outcomes were found, we re-ran our analyses after rescaling and standardisation of the independent variables to obtain a measure of the sizes of the fixed effects. We rescaled the predictors by subtracting the mean value of each continuous independent variable from their respective raw value and divided by two standard deviations. By dividing by two standard deviations the interpretation of the regression coefficients of continuous independent variables is directly comparable to that obtained for the untransformed binary predictors because a binary variable -with equal probabilities- has a mean of 0.5 and a standard deviation of 0.5 (Gelman 2008). In all analyses, we used study-specific weights to account for the disproportionate attrition of participants in MCS. In line with a Bonferroni correction for critical *p*-values we accounted for multiple testing in the models by considering *p*-values of <0.01 as significant. Analyses were run using Stata/SE 15.1 (StataCorp 2011) and MLwiN 3.02 (Charlton et al. 2018).

## Results

### Descriptive Statistics

The baseline (age 7) characteristics of children in the analytic sample (*N* = 4794) and the percentage of missing data across variables are summarised in Table 1. At age 7, children (51% male) were on average 86.72 months old (SD = 2.92), with average internalising and externalising scores of 2.78 (SD = 2.80) and 4.72 (SD = 3.50), respectively. The average KS1 score of the analytic sample was 5.57 (SD = 2.83). The majority of children comprising the analytic sample were White (*N* = 3921; 82%), lived with both biological parents (*N* = 3486; 73%) and did not have SEN (*N* = 3450; 72%). Moreover, 18% (*N* = 798) had mothers with a university degree and 16% (*N* = 648) were eligible for FSM. In comparison, the children in the non-analytic sample (those in schools outside England and those in English private schools; *N* = 3380) had lower mean internalising (M = 2.42, SD = 2.63) and externalising (M = 4.37, SD = 3.48) scores (both *p*-values <0.001), and mothers with lower levels of psychological distress (M = 2.83, SD = 3.68, *p* < 0.001). They were also more likely to have mothers with a university degree (22%; *p* < 0.001), less likely to have SEN (75%; *p* = 0.02) and more likely to be White (95%; *p* < 0.001), reflecting the higher proportion white in the population outside England. However, there was no evidence for a difference between the two groups regarding their mean age, gender or the proportion living with both biological parents (all *p*-values>0.05).

**Table 1:** Baseline sample (age 7, in around 2008) and school characteristics (in January 2008) of the analytic sample^*^ (*N* = 4794) (unweighted data)

Continuous variables	Mean	Standard Deviation	% Missing data
Child’s internalising problems	2.78	2.80	2
Child’s externalising problems	4.72	3.50	2
Child’s average Key Stage 1 score	5.52	2.89	13
Child’s age (months)	86.72	2.92	0
Maternal psychological distress	3.15	3.82	4
School size (deciles)	6.82	2.70	2
School’s pupil teacher ratio (deciles)	6.19	2.67	2
School’s proportion of native English speakers (deciles)	2.58	1.19	2
School’s proportion of free school meal eligibility (deciles)	5.51	2.79	6
School’s proportion of children with special educational needs (without statement) (deciles)	5.38	2.81	2
School’s average Key Stage 1 score (deciles)	5.57	2.83	9
School’s ethnic heterogeneity	5.61	2.78	21
Categorical variables	N	%	% Missing Data
Gender, female	2370	49	0
Child’s mother has university degree	798	18	5
Child eligible for free school meals	648	16	13
Child does not have special educational needs	3450	72	0
Child’s ethnicity			0
White	3921	82	
Mixed	161	3	
Indian	142	3	
Pakistani	251	5	
Bangladeshi	78	2	
Other Asian	41	1	
Black Caribbean	60	1	
Black African	94	2	
Other Black	15	0	
Chinese	6	0	
Other ethnic group	24	1	
Child lives with both biological parents	3486	73	0

^*^See flow chart (Fig. 1)

### Bivariate Associations between the Main Measures

Table 2 summarises the bivariate associations between the school compositional measures. The Pearson’s correlation coefficients were all statistically significant. The strongest associations were between ethnic heterogeneity and proportion of native English speakers (r = −0.78, *p* < 0.01), between average KS1 scores and proportion of children with FSM eligibility (r = −0.63, *p* < 0.01) and between proportion of children with SEN and proportion of children with FSM eligibility (r = 0.55, *p* < 0.01).

**Table 2:** Pearson’s correlation coefficients between school characteristics at baseline (age 7)

	1.	2.	3.	4.	5.	6.	7.
1. School size (deciles)	1.00						
2. School’s pupil teacher ratio (deciles)	0.40*	1.00					
3. School’s proportion of native English speakers (deciles)	−0.31*	−0.04*	1.00				
4. School’s proportion of free school meal eligibility (deciles)	0.06*	−0.30*	−0.35*	1.00			
5. School’s proportion of children with special educational needs (without statement) (deciles)	0.04*	−0.17*	−0.20*	0.55*	1.00		
6. School’s average Key Stage 1 score (deciles)	−0.14*	0.20*	0.26*	−0.63*	−0.54*	1.00	
7. School’s ethnic heterogeneity	0.21*	0.03*	−0.78*	0.25*	0.11*	−0.13*	1.00

*p < 0.01

### School Compositional Characteristics and Children’s Internalising Problems

Table 3 presents the results of MLMs examining the relationship between school compositional characteristics and internalising problem trajectories at ages 7, 11 and 14 years. All the regression coefficients presented are unstandardised, unless otherwise stated. Before adjusting for covariates (Model A), attending a school with a higher proportion of children with FSM eligibility, a larger share of children with SEN, and a lower average KS1 score was independently associated with increased levels of internalising problems at the intercept (around age 11 years).^1^ These associations did not, however, survive adjustments for individual and family characteristics (Model B). The cross-level interactions for FSM eligibility, SEN status and academic performance were also not significant (Model C) suggesting that the school’s composition did not alter the child’s likelihood of following their expected, based on their own characteristics, trajectory of internalising problems. In the fully adjusted model, males, children without SEN, those showing better academic performance, those with less distressed mothers and those living with both biological parents had lower levels of internalising problems at around age 11. The VIF in Model B, which included all the covariates (but not the interactions), ranged from 1.02 to 3.33 (mean VIF = 1.64) suggesting absence of multicollinearity.

**Table 3:** Crude and adjusted unstandardised regression coefficients of multilevel models examining the relationship of school composition and individual and family characteristics with internalising and externalising problem trajectories at ages 7 to 14

	Internalising problems			Externalising problems		
	Model A^a^	Model B^b^	Model C^c^	Model A^a^	Model B^b^	Model C^c^
	Coeff. (SE)	Coeff. (SE)	Coeff. (SE)	Coeff. (SE)	Coeff. (SE)	Coeff. (SE)
Fixed effects						
Constant	2.66 (0.35)*	3.47 (0.34)*	3.46 (0.39)*	3.57 (0.45)*	6.05 (0.42)*	5.89 (0.46)*
School characteristics						
School size (deciles)	−0.00 (0.02)	0.00 (0.01)	0.00 (0.01)	−0.00 (0.02)	0.01 (0.02)	0.01 (0.02)
School’s pupil teacher ratio (deciles)	0.01 (0.02)	0.00 (0.01)	0.00 (0.01)	0.00 (0.02)	−0.00 (0.02)	−0.00 (0.02)
School’s proportion of native English speakers (deciles)	0.02 (0.06)	0.05 (0.05)	0.05 (0.05)	0.09 (0.07)	0.06 (0.07)	0.06 (0.07)
School’s proportion of free school meal eligibility (deciles)	0.06 (0.02)*	0.02 (0.02)	0.02 (0.02)	0.14 (0.02)*	0.09 (0.02)*	0.09 (0.02)*
School’s proportion of children with special educational needs (without statement) (deciles)	0.06 (0.02)*	0.03 (0.01)	0.05 (0.05)	0.03 (0.02)	0.00 (0.02)	0.05 (0.06)
School’s average Key Stage 1 score (deciles)	−0.06 (0.02)*	0.00 (0.02)	−0.00 (0.03)	−0.08 (0.02)*	0.04 (0.02)	0.05 (0.3)
School’s ethnic heterogeneity	−0.01 (0.02)	0.00 (0.02)	0.00 (0.02)	0.01 (0.03)	0.02 (0.03)	0.05 (0.03)
Individual and family characteristics						
Age in years (centred at age around 11 years)	0.13 (0.01)*	0.13 (0.01)*	0.13 (0.01)*	−0.05 (0.01)*	−0.05 (0.01)*	−0.05 (0.01)*
Gender, female	–	0.32 (0.06)*	0.32 (0.06)*	–	−0.80 (0.07)*	−0.79 (0.07)*
Child does not have special educational needs	–	−1.13 (0.09)*	−1.07 (0.18)*	–	−1.19 (0.11)*	−1.04 (0.21)*
Child’s mother has university degree	–	−0.06 (0.09)	−0.06 (0.09)	–	−0.42 (0.10)*	−0.42 (0.10)*
Child’s average Key Stage 1 score		−0.11 (0.02)*	−0.12 (0.03)*		−0.25 (0.02)*	−0.24 (0.03)*
Maternal psychological distress	–	0.19 (0.01)*	0.19 (0.01)*	–	0.17 (0.01)*	0.17 (0.01)*
Child eligible for free school meals	–	0.11 (0.12)	0.10 (0.20)	–	0.21 (0.14)	0.20 (0.28)
Child’s ethnicity	–			–		
White		Ref	Ref		Ref	Ref
Mixed		−0.16 (0.18)	−0.16 (0.18)		−0.30 (0.22)	−0.30 (0.22)
Indian		0.01 (0.20)	0.01 (0.20)		−0.33 (0.21)	−0.33 (0.21)
Pakistani		0.32 (0.17)	0.32 (0.17)		−0.23 (0.19)	−0.22 (0.19)
Bangladeshi		0.37 (0.25)	0.38 (0.25)		−0.69 (0.29)	−0.69 (0.29)
Other Asian		0.08 (0.33)	0.08 (0.33)		−0.30 (0.39)	−0.29 (0.39)
Black Caribbean		−0.10 (0.35)	−0.10 (0.35)		−0.89 (0.35)	−0.89 (0.35)
Black African		−0.57 (0.22)	−0.57 (0.22)		−1.33 (0.27)*	−1.33 (0.27)*
Other Black		0.67 (0.50)	0.67 (0.50)		−0.44 (0.63)	−0.45 (0.63)
Chinese		−0.10 (0.74)	−0.10 (0.74)		0.23 (1.09)	0.23 (1.09)
Other ethnic group		0.00 (0.47)	0.00 (0.47)		−0.12 (0.53)	−0.12 (0.53)
Child lives with both biological parents	–	−0.39 (0.09)*	−0.39 (0.09)*	–	−0.57 (0.10)*	−0.57 (0.10)*
Cross-level interactions						
School’s proportion of free school meal eligibility (deciles) X Child eligible for free school meals	–	–	0.00 (0.03)	–	–	0.00 (0.03)
School’s proportion of children with special educational needs (without statement) (deciles) X Child does not have special educational needs	–	–	−0.01 (0.03)	–	–	−0.03 (0.03)
School’s average Key Stage 1 score (deciles) X Child’s average Key Stage 1 score	–	–	0.00 (0.00)	–	–	−0.00 (0.00)
Random effects						
Level 2 (child) intercept variance (SE)	3.98 (0.17)*	2.72 (0.13)*	2.72 (0.13)*	6.40 (0.21)*	4.13 (0.16)*	4.13 (0.16)*
Slope variance (SE)	0.00 (0.00)	0.00 (0.00)	0.00 (0.00)	0.00 (0.00)*	0.00 (0.00)*	0.00 (0.00)*
Covariance (SE)	0.01 (0.00)*	0.01 (0.00)*	0.01 (0.00)*	−0.00 (0.00)	−0.00 (0.00)	−0.00 (0.00)
Level 1 (occasion) intercept variance (SE)	5.07 (0.21)*	5.06 (0.19)*	5.06 (0.19)*	5.01 (0.21)*	4.96 (0.20)*	4.96 (0.20)*

All models were adjusted for the stratified design of MCS (regression coefficients of the strata are not shown in the table for parsimony)

*p < 0.01

^a^Model A. Model adjusted for school compositional characteristics

^b^Model B. Model A + adjustments for individual and family characteristics

^c^Model C. Model B + cross-level interactions between school- and the equivalent individual-level characteristics

### School Compositional Characteristics and Children’s Externalising Problems

**Fig. 2:**
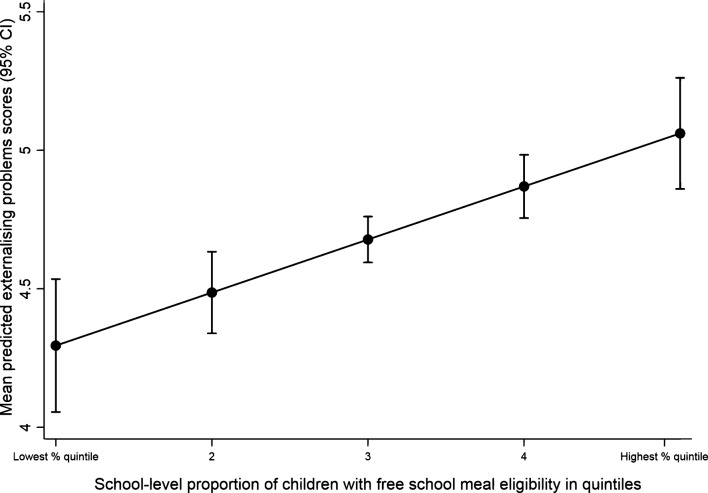
Linear predictive margins of externalising problem scores (95% CI) by quintile of school-level proportion (%) of children with free school meal eligibility (Model C)

Table 3 also presents the results of MLMs of the relationship between school compositional characteristics and externalising problem trajectories at ages 7, 11 and 14 years. In the baseline model (Model A), attending a school with a higher proportion of children eligible for FSM and lower average academic performance at age 7 was associated with increased levels of externalising problems at around age 11. School-level FSM eligibility - but not school-average academic performance - retained its significant association with externalising problems after adjustments for children’s individual and family characteristics (Model B). The cross-level interactions for FSM eligibility, academic performance and SEN status were not significantly associated with externalising problems (Model C). In this, fully-adjusted, model, females, children without SEN, those with more educated and less distressed mothers, those with better academic performance and those living with both biological parents had lower levels of externalising problems at around age 11. Figure 2 illustrates the significant association between school-level FSM eligibility and externalising problems in the fully adjusted model (Model C). To aid interpretation, we transformed the proportion of FSM eligibility into quintiles and plotted the linearly predicted margins of externalising problem scores across the five FSM-eligibility quintiles.

Moreover, in order to elucidate the relative importance of the proportion of FSM eligibility for the trajectories of internalising problems compared to the remaining individual level characteristics we re-ran this final model after re-scaling the continuous variables and dividing by two standard deviations. The results of this model suggest that, as expected, maternal psychological distress (b = 1.28, SE = 0.09), academic performance (b = −1.35, SE = 0.20) and SEN status (b = −1.03, SE = 0.20) had the largest effect sizes. The effect size of school-level FSM eligibility (b = 0.52, SE = 0.13) was comparable to the ones yielded for gender (b = −0.79, SE = 0.07), maternal education (b = −0.41, SE = 0.10) and living with both biological parents (b = −0.59. SE = 0.10).

### Sensitivity Analyses

As school poverty can simply reflect the level of socio-economic disadvantage of the area, in turn associated with mental health at least in adults, we examined neighbourhood and school poverty effects simultaneously. We therefore re-ran the fully-adjusted models for internalising and externalising problems after controlling for poverty in the child’s residential neighbourhood [Lower layer Super Output Area (LSOA)], measured using the 2004 Index of Multiple Deprivation (IMD) income deprivation score.^2^ In these new models, neighbourhood poverty had a disadvantageous effect on internalising problems (b = −0.04, SE = 0.02, *p* = 0.01), but a non-significant effect on externalising problems (b = −0.03, SE = 0.02, *p* = 0.15). In the externalising problems model, the effect of school poverty remained significant (b = 0.07, SE = 0.02, *p* = 0.002).

We also attempted to address an important study limitation: our inability to account for group level random effects due to the insufficient clustering of children within schools; as discussed, in the analytic sample 66% of schools provided information for a single MCS child, and the average MCS pupil count within a single school was 2.1. There have been recent attempts to tackle the problem of sparseness of lower-level units per higher level whereby lower-level units are grouped together into larger “synthetic” groups, yielding a larger number of cases per higher-level. However, such clustering strategies introduce artificial within-group heterogeneity by grouping together units which may differ on a host of unobserved characteristics (Clarke and Wheaton 2007). In light of this, we treated school-level characteristics at the same level as individual level characteristics in our analysis thus far. Nonetheless, we also tested our approach empirically. First, having added a third level for ‘school’, we ran two multilevel models – one for each of internalising and externalising symptoms - which accounted for MCS’s complex design but did not include any additional explanatory variables. Using this model specification, we calculated the intraclass correlation coefficient (ICC) to estimate the proportion of variance in internalising and externalising symptom trajectories that is explained by commonalities in children attending the same school. The results of the model using internalising problems as the outcome suggested that between-school variation accounted for a mere 1.7% of the variation in the outcome, yet the variance in the outcome due to children clustering within-school was not statistically significant (variance = 0.16; SE = 0.09; *p* = 0.06). Using externalising problems as the outcome, the ICC was slightly higher at 4.7% and the variance in the outcome due to children clustering within-school reached statistical significance (variance = 0.55; SE = 0.13; *p* < 0.01). Following up on this significant finding, we ran a fully adjusted multilevel model for externalising problems including the full list of covariates (Model C). The results of this model remained materially unchanged compared to the estimates (presented earlier) from the model which did not take into account the clustering of pupils within schools. These findings suggest that, due to the low degree of clustering of children within schools in MCS, considering the nesting of pupils within school adds an unnecessary layer of complexity to the multilevel models without impacting on the estimates of the regression coefficients.

## Discussion

This is the first study to examine the roles of several primary school composition and cross-level interactions between school composition and child background in trajectories of externalising and internalising problems from mid-childhood to mid-adolescence. The results suggest that, even after adjustments for children’s individual and family characteristics, children attending schools with higher proportions of FSM-eligible pupils had more externalising problems throughout childhood and adolescence. If these associations were causal, they would suggest that interventions aiming to reduce socioeconomic inequalities in schools (for example by pursuing policies that work against the segregation of pupils and schools according to SES) have the potential to improve pupils’ internalising and externalising problems and, therefore, alleviate some of the burden associated with poor psychological functioning. Nonetheless, we note that some of the individual and family factors we considered appeared to have larger associations with a child’s internalising and externalising problems. These included child’s special educational needs status and academic performance, maternal psychological distress and family structure. Thus, our study provides further support for the key role of these factors, well-established in the extant literature, in child mental health (Flouri et al. 2018; Oldfield et al. 2017; Weeks et al. 2016). It also provides more evidence about the developmental course of these child mental health difficulties in the general school population from mid-childhood to mid-adolescence. As can be seen in our fully adjusted models, age (centred at around 11 years) had a significant positive effect on the average internalising problem trajectory and a significant negative effect on that for externalising problems. This suggests that, as children reach puberty and enter secondary school, their internalising symptoms increase while their externalising problems (hyperactivity and conduct problems) decrease, in line with what previous literature supports (Angold et al. 1998; Le and Stockdale 2011).

Previous evidence also suggests advantageous effects of school-average academic performance and lower share of pupils with SEN on self-esteem and academic performance (Cvencek et al. 2018; Hienonen et al. 2018), both of which are closely linked with internalising and externalising problems (Van der Ende et al. 2016; Watkins and Melde 2016). Our results too showed significant associations between school-average academic performance and children’s internalising and externalising problems at around age 11 (and between share of pupils with SEN and individual children’s internalising problems), albeit only before adjustments for key individual and family level covariates. This suggests that the direct effects of these school compositional characteristics on children’s psychological outcomes are relatively weak and confounded.

Contrary to our expectations, our study findings offered little support to the theory of relative deprivation (Stouffer et al. 1949) or the Big-Fish-in-Little-Pond effect (Marsh and Hau 2003). The cross-level interactions that we examined were not significant for either internalising or externalising problems, suggesting that the effects of own academic performance, own SEN status or own FSM eligibility did not differ according to the school’s average academic performance, proportion of pupils with SEN or share of pupils on FSM. One possibility is that, at primary school, children are not aware of their school’s academic reputation, as the 15 year olds studied by Marsh and Hau (2003) may have been. Another possibility however is that these null findings, in particular the ones pertaining to cross-level interactions between school-level and individual academic performance,^3^ are due to lack of statistical power. Until studies with more power to detect such interaction effects are carried out, we must treat these findings as exploratory.

From the point of view of planning public health interventions, it is important to emphasise the relatively strong impact of the proportion of school FSM eligibility on the levels of externalising problems throughout childhood and adolescence. According to the results of this study, a reduction in school-level FSM eligibility by a single decile is associated with a reduction of 0.1 points on the hyperactivity scale of the SDQ. Albeit this might not appear as a strong effect, it should be interpreted in light of evidence that school-level factors are, generally, weaker predictors of outcomes than individual-level factors (Welsh et al. 1999). In fact, the effect size of school FSM eligibility was comparable to the ones obtained for established individual level risk factors of externalising problems, including low parental education (Huisman et al. 2010) and not living with both biological parents (Luoma et al. 1999). Of course this finding is not to suggest that schools should exhibit favouritism towards more affluent pupils at the selection stage in order to reduce the proportion of pupils eligible for FSM. Rather our findings provide encouraging evidence that interventions targeting poverty at the community level might prove to be effective in reducing externalising problems among pupils of such areas by lowering the number of FSM eligible pupils in local schools. Our findings also suggest that closer monitoring of pupils in schools with a high proportion of FSM eligibility is warranted in order to identify and target externalising problems at an early stage.

Our study has several strengths. The data came from the largest UK birth cohort and covered a wide range of school, family and individual characteristics that we examined in relation to externalising and internalising problems. We also used state-of-the-art statistical procedures to impute missing data and run the analyses. Additionally, by considering simultaneously characteristics at both individual and school levels, we avoided committing the ecological fallacy, whereby inference occurs at the group level (school, in this case), but is actually attributable to confounding by individual factors (Snijders and Bosker 1999). Finally, we tested the effect of cross-level interactions, which has been largely neglected in the extant literature of ‘school effects’. By doing so, we were able to test the role of the school’s composition in changing an individual pupil’s likelihood of following the path of psychological functioning that would have been expected on the basis of their individual and family characteristics.

Nonetheless, our study has limitations too, and our results should be interpreted with these caveats in mind. First, the analytic sample comprised a relatively disadvantaged group of children, which compromises the external validity of our study. Second, we did not have data on the children’s secondary schools or on the primary school at age 11 where this had changed. Third, we acknowledge that FSM is not always a good proxy for socio-economic disadvantage because there is evidence that significant numbers of children can experience socio-economic disadvantage of different forms (Ilie et al. 2017; Taylor 2018). Nonetheless, as Taylor (2018) suggests, FSM eligibility comes very close to identifying a disadvantaged group of children. Fourth, we did not control for psychological functioning prior to the study period, i.e. before age 7. Prior differences in internalising and externalising symptoms might have affected the trajectories of internalising and externalising symptoms followed across childhood and adolescence, over and above the effect of the covariates we considered. Fifth, our MCS-based sample did not have enough clustering at school-level, and therefore we may not have captured the ‘true’ between-school differences in children’s internalising and externalising problems (Welsh et al. 1999). Unlike surveys about school effects which normally recruit when the children are already clustered in schools, MCS is a longitudinal survey which recruited cohort members in infancy. Although the cohort had been tightly clustered in neighbourhoods at the initial sample, the lack of clustering in primary schools reflects both residential mobility and a degree of parental choice of school even for those who have not moved. A unique strength of our study however was that MCS can be linked to school-level data from administrative sources, and also that it has detailed, longitudinal information about family background, not necessarily available in school-based samples. Finally, we did not estimate an effect of ethnic density because of the high level of missingness in the data available. Ethnic density might have been an important omission in our list of covariates because it has been shown to promote school belonging (Benner et al. 2008), in turn predicting socio-emotional adjustment (Georgiades et al. 2013). In the absence of an ethnic density measure, we also could not estimate cross-level interactions between individual ethnicity and school ethnic composition. Individuals can feel different if they are ‘mismatched’ in ethnicity, an important component of the self-concept (Phinney 1990). For example, in neighbourhoods with more ethnic diversity there is less sense of belonging (Putnam 2007). Relatedly, there is a long history of research (Faris and Dunham 1939) usually showing, on average, worse mental health among ethnic minorities who live in neighbourhoods with a low proportion of people of their own ethnic group (Shaw et al. 2012).

## Conclusions

In this study on 4794 children of the MCS we examined the role of primary school composition in children’s trajectories of internalising and externalising problems at ages 7 to 14 years. Our findings suggest that children in schools with a higher proportion of pupils on free school meals and those in schools with poorer academic performance have more internalising and externalising problems throughout childhood and adolescence, while those attending schools with a higher proportion of pupils with special educational needs have more internalising problems only. However, once family and individual characteristics are taken into account, only the association between attending a school with a higher proportion of pupils on free school meals and own externalising problems remains significant. Of the individual and family characteristics considered, lower academic performance, having special educational needs, having a distressed mother and not living with both biological parents are associated with more internalising and externalising problems. If the associations described are causal they suggest that interventions targeting schools with less affluent overall intakes, as well as children with these individual and family characteristics, have the potential to reduce children’s internalising and externalising problems.

## Footnotes

1 As a sensitivity analysis we re-ran all MLMs with age centred at 7.2 and 14.3 years. The results remained materially identical, hence the effects of the predictors on the outcomes at age 11 also apply to assessments of internalising and externalising problems at ages –around- 7 and 14 years.

2 In deciles. Note: LSOAs are small geographical units built from groups of Census Output Areas (typically 4–6) and are constrained by the boundaries of the Standard Table wards used for Census outputs. They have, on average, 1500 residents and are generally smaller than wards.

3 Own and school’s average academic performance were each measured in deciles. Their interaction results in a 10 × 10 contingency table (81 degrees of freedom). The power of a χ^2^ test for this interaction is high only when trying to detect medium and large (> = 0.2 power > 99%), but not small (< = 0.1, power < 80%), effect sizes at a 0.01 level of significance.
